# Notes and News

**Published:** 1901-04-20

**Authors:** 


### Notes and News.

### HOSPITAL MEETINGS AND FESTIVALS.

Metropolitan Hospital.—Anniversary Festival, Monday, April 22nd, at the Whitehall Rooms, Hotel Metropole, at 7 p.m.

East London Hospital for Children.—Festival dinner. Tuesday, April 30, at 7 for 7.30 p.m., at the Hall of the Worshipful Company of Grocers, Prince's Street, Bank.

Royal Albert Orphan Asylum, Bagshot.—Twelfth Public Dinner, Monday, April 29th, at the Whitehall Rooms, Hotel Metropole, 7 p.m. for 7.15 p.m. President, the Right Hon. Viscount Duncannon, C.B.

Royal Hospital for Incurables, Putney.—Annual Dinner, Tuesday, May 14th, at the Hotel Cecil, 6.30 p.m. for 7 p.m. President, the Right Hon. Lord Tweedmouth.

The honorary secretaries of the Prince of Wales's Hospital Fund for London are authorised to state that His Majesty the King has graciously consented to act as Patron of this Fund, and also that His Royal Highness the Duke of Cornwall and York has expressed his pleasure to accept the office of President in the place of His Majesty the King. His Majesty has intimated that in his capacity as Patron he will continue to watch over the work of the Fund with the same deep interest as hitherto.

In the Highlands of South Inverness-shire, near Kingussie, a sanatorium for consumptives is now completed. The accommodation is for twenty patients. The building faces south-east. The site is 850 feet above the sea-level; the air is bracing, pure, and dry. The surroundings consist of ten acres of woodland, which form a complete shelter to the buildings on three sides, and are intersected with suitable walks for the patients. The views are beautiful and extensive.

We recently commented upon the large infant mortality in France. But this is exceeded by recent statistics from Russia. In some districts only 50 per cent. of the children survive their first year, whilst in many others only 732 out of every 1,000 survive. Asiatic cholera alone is stated to be responsible for an annual mortality of about 385,000 Russian children, but the main factor in causing the excessive number of early deaths is considered to be irregular and unsuitable feeding, more especially amongst peasants in the country districts.

There is still a good number of small-pox patients in Paris.

The Duke of Beaufort has opened the new wards at the Bristol Eye Hospital. The extension has cost £6,800.

The French Government is proposing to send out a commission to study yellow fever, probably to Rio Janeiro. The disease has created great ravages in Senegal.

Sir Douglas Powell will deliver the Cavendish Lecture of the West London Medico-Chirurgical Society in June at the Hammersmith Town Hall. The subject of the lecture will be "Acute Cardiac Failure."

It is reported that the Japanese Government is about to try and organise a systematic and universal crusade against rats, recognising the dangerous part they play in spreading disease, more especially the plague.

Six medical men have been selected to go to South Africa for plague duty. They are Drs. G. J. Blackmore, J. Spencer Low, D. C. Rees, R. G. Kirton, W. G. Montgomery, and G. V. Worthington. Mr. D. C. Rees was late superintendent and medical tutor at the London School of Tropical Medicine.

An institute of tropical hygiene has been opened at Hamburg, close to the harbour. Fifty beds have been provided for the reception of patients suffering from tropical diseases, such as malaria and beri-beri. The institute, which belongs to the State of Hamburg, is subsidised by the Imperial Government, which retains for itself certain privileges.

A former medical practitioner, by name T. R. Allison, living in Spanish Place, Manchester Square, has been required to undertake not to circulate pamphlets of an improper nature which he had been advertising for some time past. He was bound over in his own recognisances, in £250 on each of two summonses, to appear before the Court if called for, and required to pay the costs of the prosecution, amounting to £10 10s.

There is much alarm at Port Elizabeth from the discovery of rats dead from the plague. Since the outbreak of plague in Capetown, military stores and troops have landed at Port Elizabeth. At Kalgoorlie a case of plague is reported, and a medical student contracted the disease at Michigan, America, when making some bacteriological experiments. All the ten cases of plague reported from San Francisco have proved fatal.

Without the possession of medical experience the duties of a School Board officer are exceedingly difficult. Practically he has to decide between actual and sham illness on the part of many truants. He is quite liable to grave error. This was actually the case recently at Bristol, where an officer insisted upon the attendance of a boy at school when suffering from a severe attack of bronchitis which ended fatally on his return home. It would be wiser if the school officers never enforced attendance when illness is alleged until the child had been examined by a medical man. Without this precaution an epidemic could very easily be introduced into a school, added to the injury to the individual child which might result.

